# Confined-domain crosslink-enhanced emission effect in carbonized polymer dots

**DOI:** 10.1038/s41377-022-00745-4

**Published:** 2022-03-10

**Authors:** Songyuan Tao, Changjiang Zhou, Chunyuan Kang, Shoujun Zhu, Tanglue Feng, Shi-Tong Zhang, Zeyang Ding, Chengyu Zheng, Chunlei Xia, Bai Yang

**Affiliations:** 1 State Key Laboratory of Supramolecular Structure and Materials, College of Chemistry, Jilin University, Changchun, 130012 China; 2 College of Chemical Engineering, Zhejiang University of Technology, Hangzhou, 310014 China; 3 Key Laboratory of Organ Regeneration and Transplantation of the Ministry of Education, the First Hospital of Jilin University, Changchun, 130061 China

**Keywords:** Nanoparticles, Polymers

## Abstract

Revealing the photoluminescence (PL) origin and mechanism is a most vital but challenging topic of carbon dots. Herein, confined-domain crosslink-enhanced emission (CEE) effect was first studied by a well-designed model system of carbonized polymer dots (CPDs), serving as an important supplement to CEE in the aspect of spatial interactions. The “addition-condensation polymerization” strategy was adopted to construct CPDs with substituents exerting different degrees of steric hindrance. The effect of confined-domain CEE on the structure and luminescence properties of CPDs have been systematically investigated by combining characterizations and theoretical calculations. Such tunable spatial interactions dominated the coupling strength of the luminophores in one particle, and eventually resulted in the modulated PL properties of CPDs. These findings provide insights into the structural advantages and the PL mechanism of CPDs, which are of general significance to the further development of CPDs with tailored properties.

## Abstract (web summary)

Confined-domain crosslink-enhanced emission effect was first studied to reveal the photoluminescence mechanism of carbonized polymer dots (CPDs). A general strategy was exploited to synthesize CPDs exhibiting tunable photoluminescence properties.

## Graphical abstract

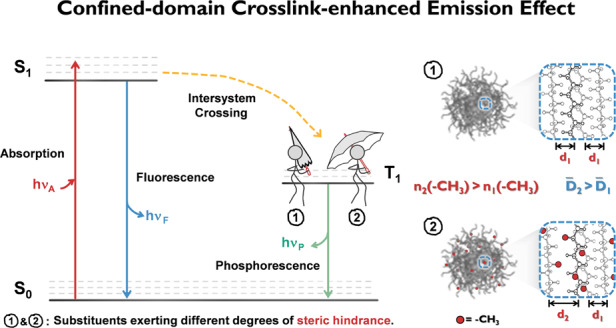

## Introduction

Carbon dots (CDs) exhibit tremendous superiority in facile fabrication and luminescence performance^1–4^. These novel nanomaterials have realized widespread applications in optoelectronic devices, biomedicine, photocatalysis, etc^5–7^. The ultimate purpose of investigations on CDs is to recognize the essence of these materials and achieve the targeted synthesis. However, it’s a tough task to modulate the properties of CDs, since the PL mechanism is still controversial^8–12^. Inspired by the mature theories of quantum dots^13–15^, graphene^16,17^, and organic molecules^18,19^, some speculations have emerged about the photoluminescence (PL) mechanisms of CDs, including carbon-core states, surface/edge states, and molecule states. Nevertheless, the PL behaviors of CDs cannot be fully clarified and controlled due to the complexity in species and structures.

Carbonized polymer dot (CPD) is an updated definition to describe the subclass of CDs with carbonized core inside and polymer structure outside^17,20,21^. Actually, the majority of CDs synthesized from molecules or polymers by “bottom-up” method should belong to CPDs. CPDs undergo the process of dehydration and crosslinking. The performances of CPDs primarily depend on the balance of polymerization and carbonization^17,22^. Yang et al. recognized the contribution of crosslinking to the generation of nanostructures and excellent performances in CPDs, and proposed the pioneering concept of crosslink-enhanced emission effect (CEE)^23,24^. An insight into the mechanism can potentially help understand the unconventional PL behaviors exhibited by the sub-luminophores in non-conjugated polymer dots (includes CPDs)^24^. Currently, novel properties and applications of CPDs based on CEE have been widely exploited^25–29^.

CEE was considered as an immobilization effect on luminophores at its initial stage, that exerted the influence by suppressing non-radiative transitions through bonding interactions. Such enhancement mode by immobilizing widely exist in various luminescent materials, like the famous aggregation-induced emission phenomena in organic molecules^30–32^. Notably, benefiting from the special nanostructure, CPDs always exhibit extra ability of PL emission, compared to similar bond-forming compounds.

From a deeper perspective, crosslinking forms a stable and compact environment for the fluorophores inside CPDs. Spatial effect in these highly-crosslinked domains (herein defined as confined-domain CEE) can greatly promote the electron-cloud overlaps and energy-level splittings in CPDs without the need to form specific chemical bonds^33,34^. Thus, it is reasonable to infer that confined-domain CEE significantly contributes to the generation of the PL in CPDs^35–38^. Investigating these interactions using appropriate methods is crucial^39^. Clarifying confined-domain CEE will further promote the development of the fundamental PL theory, and provide with a general strategy to modulate the PL of CPDs, that makes great sense.

This study aims to unambiguously identify the concept of confined-domain CEE in CPDs. A model system of CPDs possessing a well-defined structure was constructed by the “addition-condensation polymerization” strategy. We polymerized acrylic acid (AA) and methylacrylic acid (AACH_3_) in varying proportions, and subsequently synthesized CPDs from the obtained copolymers and ethylenediamine (EDA) following a hydrothermal method. The tunable content of methyl groups in copolymers was expected to bring about varying degrees of steric hindrance. Some interesting phenomena and experimental evidences demonstrated the contribution of confined-domain CEE to the PL in CPDs: (1) The spatial interactions and PL performance could be tuned by tuning the steric hindrance present in the interior of the CPDs. (2) Results from the structural characterizations demonstrated the introduction of methyl groups enlarged the spacing of chains inside nanoparticles. (3) The analysis of the femtosecond transient absorption (TA) spectral profiles revealed the existence and influence of confined-domain CEE on energy levels. 4) The theoretical calculations proved the rationality of the conjectured luminescent units and the proposed PL mechanism. This study is first where confined-domain CEE in CPDs has been experimentally studied. These results can potentially help understand the unique structural advantages of CPDs and precisely tune their PL properties.

## Results

### Construction for a model system of CPDs

Selecting a representative and appropriate model system is most important for the study of the confined-domain CEE in CPDs while considering the following points^17^: (1) Structural primitives are required for the formation of CPDs. Amino and carboxyl groups are the most common groups used for the development of CPDs. (2) Tunable spatial interactions must be present inside one particle. This can be achieved by introducing the substituents that exert varying degrees of steric effect. (3) A general hydrothermal synthesis method should be followed. (4) Avoid interference from other factors in the system, such as thermal decomposition and side reactions. (5) Investigations on the PL mechanism of CPDs should cover the entire photophysical process of both fluorescence and phosphorescence. We have previously proposed CPDs as a new class of metal-free room-temperature phosphorescence (RTP) materials. Unexpected RTP in CPDs was first realized by one-step synthesis from polyacrylic acid (PAA) and EDA^27^.

**Fig. 1:**
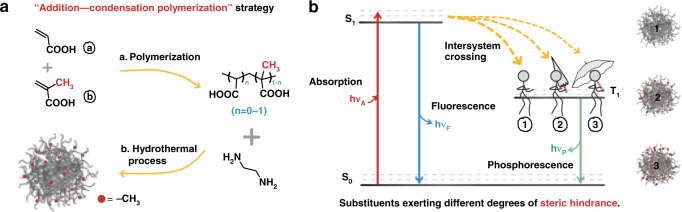
General design for studying confined-domain CEE in CPDs. **a** Schematic of the “addition-condensation polymerization” strategy. **b** Schematic Jablonski diagram of the possible PL mechanism for CPDs with substituents exerting different degrees of steric hindrance (1: no steric hindrance. 2: small steric hindrance. 3: large steric hindrance)

On the basis of comprehensive considerations, we adopted the strategy for “addition-condensation polymerization” here to further reveal the confined-domain CEE in CPDs (Fig. 1a). In this model system, AA and AACH_3_ were copolymerized in varying proportions by the radical polymerization. Subsequently, the synthesized polymers and EDA were used as the raw materials to prepare CPDs by hydrothermal method (Supporting Information). Compared to the aromatic groups, aliphatic groups were deem to exert less influence on the luminescence properties of the materials^19^. Thus, methyl groups were introduced to sterically hinder the luminescent units present inside CPDs. Varying in the content of methyl groups was expected to influence the PL properties of CPDs by modifying the spatial interactions (Fig. 1b). The purified CPDs where the AA to AACH_3_ ratios were 1/0, 1/1, 0/1 were selected as samples for further studies, respectively marked as CPDsCH_3_-1, CPDsCH_3_-2, and CPDsCH_3_-3. CPDs powders are yellowish and easy to grind finely and redissolve in water. The solutions of CPDs are always transparent, exhibiting colorless at a low concentration and yellowish at a high concentration. CPDsCH_3_-1 powder exhibits the best water solubility in all CPDs samples due to the absence of hydrophobic methyl groups (Fig. S1).

### Morphologies and PL properties of CPDs

Information on the morphologies of CPDs was gained from the transmission electron microscopy (TEM) and atomic force microscopy (AFM) techniques. Analyses of the results revealed the formation of nanoparticles in all of the cases. The TEM images of CPDs indicated the presence of well-dispersed dots with average diameters of 3.7 ± 0.63, 4.5 ± 0.66, and 5.4 ± 0.71 nm (Fig. S2). Most of the dots observed using the TEM technique were amorphous and devoid of detectable lattice fringes, indicating the internal structure of CPDs should be a crosslinked polymer network instead of a graphitized core. Besides, CPDsCH_3_-1 exhibited the most distinguishable profile and highest image contrast benefiting from the best crosslinking. The AFM images revealed the topographic heights uniformly increased as the content of methyl groups increased (Fig. S3), that agreed well with the results obtained by analyzing the TEM images.

**Fig. 2:**
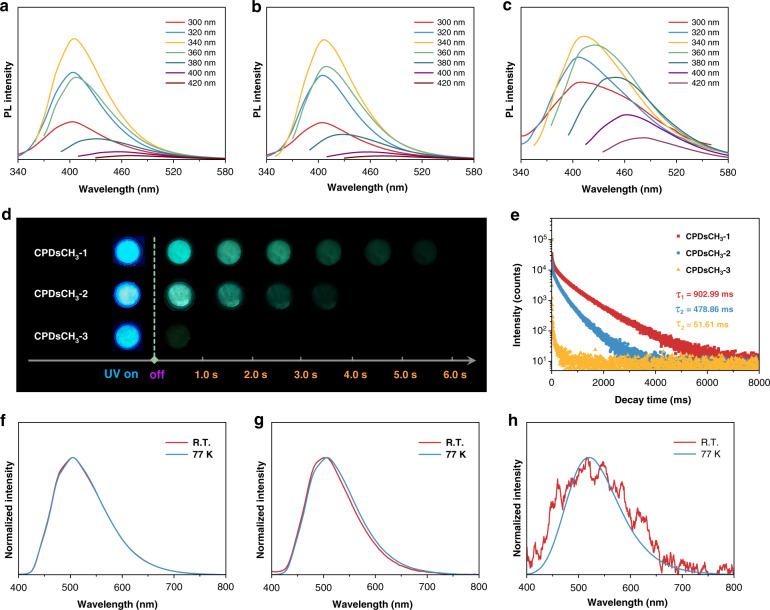
Luminescent properties of CPDs. PL spectra recorded with solution-state **a** CPDsCH_3_-1, **b** CPDsCH_3_-2, and **c** CPDsCH_3_-3. **d** Images corresponding to the delay time recorded post UV irradiation. **e** RTP decay spectral profiles. Normalized afterglow spectra of **f** CPDsCH_3_-1, **g** CPDsCH_3_-2, and **h** CPDsCH_3_-3 at room temperature (R.T.) and 77 K

Luminescent behaviors of CPDs in different states were thoroughly investigated to further reveal the difference in PL properties. Aqueous solutions of CPDs emitted intense blue fluorescence. However, they did not exhibit the property of phosphorescence. All the CPDs exhibited characteristic excitation-dependent fluorescence. The optimal excitation and emission occurred at ~340 and ~410 nm, respectively (Fig. 2a–c). Similar PL decay curves were recorded when experiments were carried out with CPDsCH_3_-1 and CPDsCH_3_-2 (Fig. S4). The decay curves fit the lifetime with two similar exponential components (Table S1). The lifetime of CPDsCH_3_-3 was greatly shortened due to the increased numbers of non-radiative transitions occurring in the presence of excess methyl groups. The fluorescence features of solid- and solution-state CPDs were remarkably similar, such as emission and excitation wavelength and nature of the photophysical processes (Figs. S5, S6, Table S2). Interestingly, the CPDs powders exhibited significant differences in the afterglow (RTP) when the UV illumination was turned off (Fig. 2d). The decay of RTP was also recorded by videos (Supporting Movies). CPDsCH_3_-1 exhibited the most intense emission and the longest delay time (τ_1_ = 902.99 ms; followed by CPDsCH_3_-2: τ_2_ = 478.86 ms), while CPDsCH_3_-3 exhibited almost no RTP (Fig. 2e).

Ultralow temperature can suppress the non-radiative transition to the greatest extent, and completely exhibit the emissive ability of materials. The afterglow spectral profiles (Fig. 2f–h) were analyzed to understand the emission behavior of CPDs at room temperature or 77 K (in liquid nitrogen). The phosphorescence of CPDs at 77 K indicated that PL centers were formed in all three samples^27^. Notably, the two curves of CPDsCH_3_-1 perfectly coincided with each other at room temperature and 77 K, indicating a most stable spatial interaction. This property could be attributed to the compact confined domains of the particles. These results were also validated by analyzing the RTP decay spectral profiles at 77 K (Fig. S7). Summarizing the above discussions, the confined-domain CEE can potentially modify the PL properties of CPDs (CPDsCH_3_-1 and CPDsCH_3_-2), and ultimately arise the changes in energy levels (CPDsCH_3_-3).

### Studies on the PL origin and bonding mode in CPDs

**Fig. 3:**
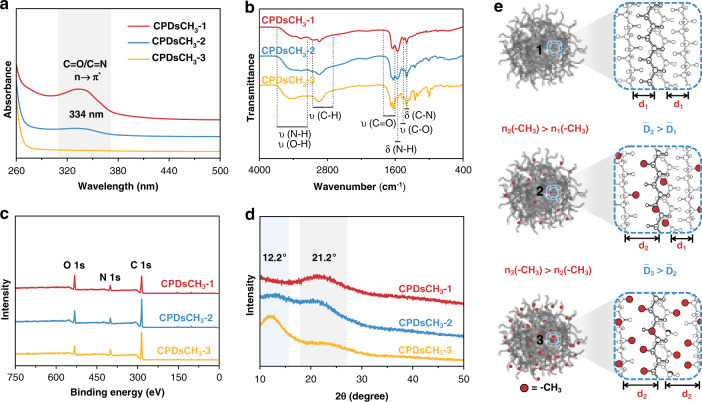
Effect of the methyl groups on the structures of CPDs. **a** UV–vis absorption spectral profiles. **b** FTIR spectral profiles. **c** XPS spectral profiles. **d** XRD patterns. **e** Schematic of the relationship between the methyl groups and the internal structure of CPDs (d_1_: the spacing of the original skeleton inside CPDs without methyl groups. d_2_: the spacing of the stretched skeleton inside CPDs by methyl groups. D_1_: average skeleton spacing of CPDsCH_3_-1. D_2_: average skeleton spacing of CPDsCH_3_-2. D_3_: average skeleton spacing of CPDsCH_3_-3)

The samples were characterized to confirm the formation of the target CPDs containing varying numbers of methyl units. Results from the analysis of the normalized ultraviolet-visible (UV-vis) absorption spectra (Fig. 3a) revealed that the peak focused at approximately 334 nm could be assigned to the n→π* transition of the C = O/C = N bonds^4,27^. The absorption of carbon skeleton in the short-wavelength region strengthened as the content of methyl groups increased, which made the peak at 334 nm less obvious but still existed. In the Fourier transform infrared (FTIR) spectra (Fig. 3b), the presence of characteristic N-H (3350 and 1556 cm^−1^), C = O/C = N (1645 cm^−1^), and C-N (1395 cm^−1^) vibrational peaks confirmed the formation of amide/imide bonds in CPDs^27,40^. A sharp peak at 1632 cm^−1^ emerged in the region where vibrational peaks corresponding to the vibration of the C = O/C = N groups are observed, that was most prominent in CPDsCH_3_-3. This peak might have some impact on amide/imide-based luminescence centers, exactly corresponding to the PL behaviors as discussed above. The peaks corresponding to the C-H stretching vibrations appeared at approximately 2945 cm^−1^, that presented a regular change from CPDsCH_3_-1 to CPDsCH_3_-3 as expected.

### Effect of methyl groups on the structures of CPDs

The X-ray photoelectron spectroscopy (XPS) technique was used to study the surface-bonding properties of all three samples in detail (Fig. 3c, Fig. S8)^22^. The C 1 s band revealed the presence of C-C/C = C (284.6 eV), C-O/C-N (285.9 eV), and C = O (287.8 eV) units. The N 1 s band was deconvoluted into two peaks at 399.5 and 401.1 eV, corresponding to the C-N and N-H units, respectively. The O 1 s band exhibited two peaks centered at 531.3 and 532.5 eV, corresponding to the C = O and C-O units, respectively. The CPDs exhibited same bonding characteristics but different proportions. Quantitative analysis from XPS measurement (Table S3), showed CPDs possessed similar N content (related to luminophore) and increasing C content (related to steric hindrance). These results were also confirmed by the global element analysis experiments (Table S4), that fit well with the above-described structural features of expected CPDs.

The nuclear magnetic resonance (NMR) spectroscopy (^1^H and ^13^C) technique was used to further investigate the structures of the CPDs (Figs. S9, S10). All the signals observed in the ^1^H and ^13^C NMR spectra could be assigned according to the simulated chemical shifts^40,41^. The NMR signal at ~1.0 ppm in the ^1^H NMR spectra could be attributed to the methyl protons. The signals observed in the range of 20.0~30.0 ppm in the ^13^C NMR spectra also proved the existence of the methyl groups. The results can be summarized into three points: (1) All the CPDs bear amide (or imide) groups as the PL centers. (2) Methyl groups were successfully introduced into CPDs. (3) The number of methyl groups could be adjusted.

The X-ray diffraction (XRD) and high-resolution TEM (HR-TEM) techniques were used to directly explore the influence of the methyl groups on the internal structure of the CPDs. The XRD patterns revealed that the two major diffraction peaks were located at 12.2° and 21.2° (Fig. 3d). The relationship between the introduced methyl groups and internal structure of CPDs was schematically depicted in Fig. 3e based on the XRD data. CDs usually exhibit a broad diffraction peak at 20~25°, which can be assigned to the carbon skeleton inside particles formed by crosslinking and/or carbonization processes (d_1_ in Fig. 3e)^4,11,40^. It was known that d increases as θ decreases under constant n and λ (Bragg’s equation: 2dsinθ = nλ). The peak at 12.2° represented the formation of sub-domains with expanded interplanar spacing caused by methyl groups (d_2_ in Fig. 3e). As the content of methyl groups increased (from CPDsCH_3_-1 to CPDsCH_3_-3), the peak at 12.2° became more prominent. Thus, it could be inferred that the introduction of the methyl groups resulted in increased steric hindrance. The average distance between the crosslinked polymer skeletons correspondingly enlarged (D in Fig. 3e). Lattice fringes with unusual spacing values of 0.358 and 0.270 nm were observed when the HR-TEM images of CPDsCH_3_-3 were analyzed (Fig. S11). These values were significantly larger than the characteristic spacings of graphene, including 0.21 nm for (100) planes and 0.34 nm for (002) planes, that should stem from the lattice distortion of certain carbonized skeleton due to the existence of methyl groups^9,11,14^. Methyl groups primarily affected the spatial distance between the luminescence units in CPDs.

Thermograms by thermo gravimetric analyzer (TGA) showed two major thermal events occurring at 150~500 °C (Fig. S12), corresponding to the decomposition of crosslinked polymer networks (T_1_) and carbon skeleton (T_2_). From CPDsCH_3_-1 to CPDsCH_3_-3, the decreased T_1_ but increased T_2_, respectively represented a weakened crosslinking degree and a enhanced skeleton strength with the introduction of methyl groups. TGA results exactly matched our conjecture on the internal composition and structure of CPDs.

### Spatial interactions in the interior of CPDs

**Fig. 4:**
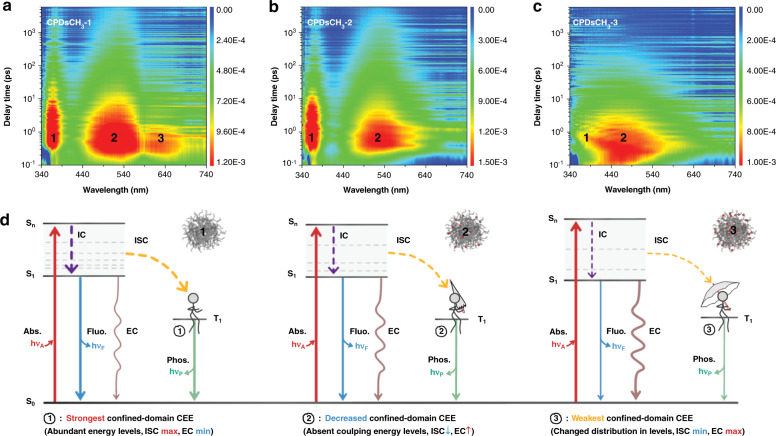
Spatial interactions observed in the interior of CPDs (confined-domain CEE). Two-dimensional pseudo-color TA maps of **a** CPDsCH_3_-1, **b** CPDsCH_3_-2, and **c** CPDsCH_3_-3 (excitation: 320 nm; probed in the range of 340~740 nm; scan delay times in the range of 0.1 ps~6 ns). **d** Schematic effect of confined-domain CEE on the energy levels of CPDs (IC: internal conversion, Abs.: absorption, Fluo.:fluorescence, Phos.: phosphorescence)

Assessing abstract spatial interactions is a tough task. Confined-domain CEE, as a PL mechanism, fundamentally influences the energy levels of CPDs, that can be ultimately reflected in the PL properties of the materials. The femtosecond TA spectroscopy technique was used to obtain information on the excited-state structures of CPDs^42^. Two-dimensional pseudo-color TA maps validated the existence of the spatial interactions in CPDs (Fig. 4a–c). For Fig. 4a, b, the primary absorption domains appeared at 365 nm (TA_1_) and 525 nm (TA_2_), indicating that CPDsCH_3_-1 and CPDsCH_3_-2 exhibited similar energy-level structures at excited state. Notably, an absorption peak at 625 nm (TA_3_) was observed in the spectra recorded with CPDsCH_3_-1, but absent in the spectra recorded with CPDsCH_3_-2. This phenomenon indicated the number of transition channels in CPDsCH_3_-1 was greater than that in CPDsCH_3_-2, that could be attributed to the stronger confined-domain CEE caused by better crosslinking in the absence of methyl groups. The increased number of pathways potentially improved the transition probability including intersystem crossing (ISC). This results in the possible enhancement in absorption and/or emission, which is benefit to PL. The absorption of CPDsCH_3_-3 (Fig. 4c) was significantly different from those of CPDsCH_3_-1 and CPDsCH_3_-2. Blue-shifted TA_2_ in CPDsCH_3_-3 represented an increased energy gap, resulting from the weakened spatial interactions. Figure S13 showed the TA spectra corresponding to different time delays. The similar excited-state behaviors represented the same PL centers in all CPDs. The kinetic traces at selected wavelengths were presented in Fig. S14. Three exponent decay functions were used to globally fit the carrier relaxation dynamics of the CPDs (Tables S5–S7). The fitting results revealed that CPDsCH_3_-1 possessed the longest carrier lifetime and best PL performance. Moreover, Fig. 4d exhibited the schematic diagrams for a better description to the effect of confined-domain CEE on energy levels. Combined with the TA analysis and PL properties, CPDsCH_3_-1 exhibited the strongest confined-domain CEE, and possessed abundant sub-levels originated from intrinsic state (TA_1_ and TA_2_) and coupling (TA_3_). As illustrated, the increasing methyl groups weakened the strength of confined-domain CEE, and the impacts were reflected on the following aspects: (1) Promote non-radiative transitions, mainly referred to external conversion (EC) rather than ISC. (2) Reduce the possible sub-levels formed by coupling (CPDsCH_3_-2). (3) Change the distributions of intrinsic energy levels (CPDsCH_3_-3).

### Theoretical calculations

**Fig. 5:**
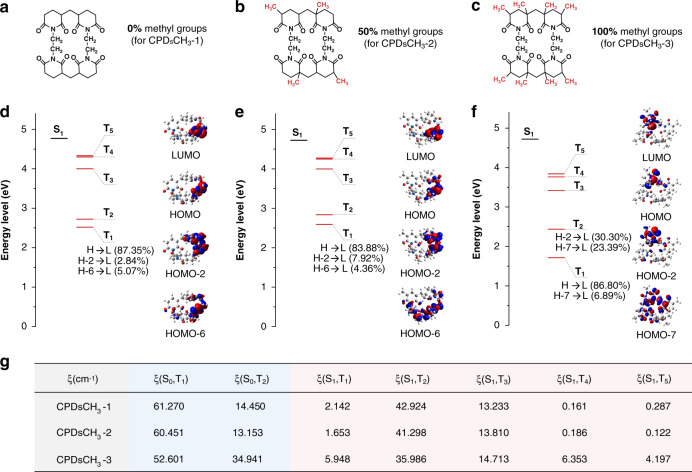
Results of theoretical calculations carried out with CPDs. Simplified luminescent units for in **a** CPDsCH_3_-1, **b** CPDsCH_3_-2, and **c** CPDsCH_3_-3. Energy-level diagrams of simplified luminescent units in **d** CPDsCH_3_-1, **e** CPDsCH_3_-2, and **f** CPDsCH_3_-3. **g** Corresponding SOC coefficients

Theoretical calculations were conducted to gain deeper insights. The effect of methyl groups on the PL of the three samples was studied. Initially, we identified the possible luminescent units present in the CPDs with varying numbers of methyl groups (Fig. 5a–c). We optimized the geometries of the simplified units in the ground and triplet states and studied the energy-level distributions. The spin-orbit coupling (SOC) coefficients were calculated to quantitatively estimate the probability of ISC and triplet emission. The results obtained from theoretical calculations agreed well with the experimental results.

On one hand, the methyl unit was found to dictate the configuration of the luminescence unit. The degree of plane distortion increased as the content of methyl groups increased, resulting in changed energy-level structures^43,44^. The radiative transition modes observed in CPDsCH_3_-3 were different from those observed in CPDsCH_3_-1 and CPDsCH_3_-2 (Fig. 5d–f). The energy of T_1_ state in CPDsCH_3_-3 was lowered to match the energy of a defect state (Table S8). The radiative transition (phosphorescence) occurred from a higher excited state (T_2_). Relevant researches have pointed out that the PL lifetime when the emission occurred from the high energy excited states was shorter than the PL lifetime when the emission occurred from the lowest excited state^45,46^. Besides, the presence of the defect states resulted in decreased QYs, that will be discussed in the following section.

On the other hand, ISC from S_1_ to triplet manifold (T_n_) was denoted as (S_1_,T_n_). ξ(S_1_,T_n_) presented the generation ability of the triplet excitons. The radiative transitions from triplet manifold (T_n_) to S_0_ was denoted as (S_0_,T_n_). ξ(S_0_,T_n_) revealed the extent of phosphorescence emission. As shown in Fig. 5g, the values of ξ(S_1_,T_n_) and ξ(S_0_,T_n_) decreased from CPDsCH_3_-1 to CPDsCH_3_-3, that was responsible for the weakened RTP performance of CPDs.

### Modulated PL performance of CPDs by confined-domain CEE

CPDs exhibiting diverse PL properties can be designed and synthesized based on the concept of confined-domain CEE. The RTP lifetimes and QYs of CPDs were continuously tuned by arousing different degrees of confined-domain CEE (Fig. S15, Table S9, Fig. S16). Besides, the values of the radiative transition rate (k_r_) and non-radiative transition rate (∑k_nr_) were calculated to reveal the transition behaviors (Table S10). The results indicated k_EC_ increased while k_r_ and k_ISC_ decreased, that further verified the above conclusions. Thus, the effect of confined-domain CEE involved multiple photophysical processes, and could accordingly modulate the emission of both fluorescence and phosphorescence. CPDs exhibiting tunable RTP lifetimes can be potentially used as smart materials to develop multi-level anti-counterfeiting technology, that is a hot field of recent study^29,47,48^.

Variability in the properties of CPDs arisen from methyl groups is relatively limited as methyl group is simple in structure. Actually, confined-domain CEE is a general strategy to modulate the PL properties. We believe that CPDs with diverse features and extensive functions can be developed by exploiting the concept of confined-domain CEE. Various substituents, such as aromatic rings and halogens, can be introduced into the structures of precursors for developing novel CPDs^49–51^.

## Discussion

In summary, we constructed a model system to study the influence of spatial interactions on PL within the confined domains of CPDs. We synthesized a series of copolymers that exerted different degrees of steric hindrance by varying the ratio of AA to AACH_3_. The prepared CPDs exhibited controllable PL properties. The TEM and XRD techniques were used to record the structural changes in CPDs caused by the introduction of methyl units. Analysis of the results obtained from the TA experiments and theoretical studies provided the information on the excited-state structures and energy-level distributions of CPDs. The results revealed that the confined-domain CEE exerted significant spatial influence on PL. The “addition–condensation polymerization” strategy could be adopted to tune the properties of the CPDs. Various factors, such as the type of the monomer precursors, the ratios of the constituent monomers, and the degrees of polymerization, could be precisely tuned to modulate the properties of the CPDs. Thus, the synthesis of CPDs can eliminate the simple choices of existing compounds as the current stage, and become more targeted. Our findings can potentially help understand the PL mechanism of CPDs, and inspire a novel synthetic design to obtain CPDs with tailored properties.

## Materials and methods

### Materials

Acrylic acid (AA), methylacrylic acid (AACH_3_), ethylenediamine (EDA), and quinine sulfate were purchased from Aladdin Chemical Co. Sulfuric acid (H_2_SO_4_, 98%) was obtained from Beijing Chemical Reagent Co. Potassium persulfate (K_2_S_2_O_8_) was purchased from Tianjin Huadong Chemical Reagent Co.

All of the chemicals were used without further purification.

### Synthesis of polymers

General preparation of polymers. The polymers were prepared by radical copolymerization of AA and AACH_3_ with 5 mg K_2_S_2_O_8_ as a radical initiator in 20 mL H_2_O under a nitrogen atmosphere at 65 °C for 8 h. A series of polymers could be synthesized by changing the ratio of AA and AACH_3_ (keep the total amount to 0.5 mL). The selected ratios (AA/AACH_3_, mL) include: 0.5/0, 0.475/0.025, 0.45/0.05, 0.425/0.075, 0.4/0.1, 0.375/0.125, 0.35/0.15, 0.325/0.175, 0.3/0.2, 0.275/0.225, 0.25/0.25, 0.225/0.275, 0.2/0.3, 0.175/0.325, 0.15/0.35, 0.125/0.375, 0.1/0.4, 0.075/0.425, 0.05/0.45, 0.025/0.475, 0/0.5.

### Synthesis of CPDs

2 mL Polymers and 100 µL EDA were dissolved in 10 mL deionized water, and mixed uniformly. Then the solution was transferred to a 25 mL poly (tetrafluoroethylene) (Teflon)-lined autoclave and heated at 200 °C for 8 h in oven. After the reaction, the reactors were cooled down to room temperature naturally. The obtained transparent buff solution was fully dialyzed in dialysis bag (500~1000 D) against deionized water for three days to remove unreacted raw materials. Solid CPDs could be obtained from the solution via freeze-drying.

### Characterization

TEM images were recorded on a JEOL JEM-2100F using ultra-thin carbon films as grids. AFM images were recorded in the tapping mode with a Nanoscope IIIa scanning probe microscope from Digital Instruments under ambient conditions. PL spectra were performed on a RF-6000 PC spectrophotometer (Shimadzu) in solution state and on FLS920 in solid state. PL lifetimes (with TCPSC technology) and emission spectra at different temperatures were collected on FLS980 (Edinburgh). RTP lifetimes were measured under the optimal excitation wavelength from a μF920H flash lamp source on FLS980 (Edinburgh). Afterglow spectra were captured by an optical fiber detector that connected to Maya 2000 Pro CCD spectrometer under 365 nm laser irradiation. UV-vis absorption spectra of solution were obtained using a 3100 UV-vis spectrophotometer (Shimadzu). IR spectra were performed with a Nicolet AVATAR 360 FT-IR spectrophotometer. XPS spectra were taken on an ESCALAB 250 spectrometer with a mono X-ray source with Al Kα excitation (1486.6 eV). NMR spectra were performed with a Bruker AVANCE NMR spectrometer (500 MHz) using D_2_O as the solvent. Element analysis was performed on Elementar Vario MICRO CUBE. XRD patterns were measured using Empyrean (PANalytical B.V.). TGA was performed on a Mettler Toledo TGA/SDTA851e instrument under N_2_ atmosphere from room temperature to 800 °C with a heating rate of 10 °C min^−1^.

The femtosecond transient absorption setup was based on a regenerative amplified Ti:sapphire laser system from Coherent (800 nm, 35 fs, 0.7 µJ cm^−2^ per pulse, and 1 kHz repetition rate), nonlinear frequency mixing techniques and the femto-TA-100 (Time-tech Spectra company). A 50% beam splitter split the 800 nm output pulse from the regenerative amplifier into two parts. The transmitted part was used to pump a TOPAS Optical Parametric Amplifier (OPA) that could generate a wavelength-tunable laser pulse (250 nm~2.5 µm) as a pump beam. The reflected part was split again into two parts. One part with less than 10% was attenuated with a neutral density filter and focused into a 2 mm thick sapphire window to generate a white light continuum (WLC) from 300~800 nm as a probe beam. The probe beam was focused by an Al parabolic reflector onto samples. After the sample, the probe beam was collimated and then focused into a fiber-coupled spectrometer with CMOS sensors and detected at a frequency of 1 kHz. The intensity of the pump pulse adopted in the experiment was controlled by a variable neutral-density filter wheel. The delay between the pump and probe pulses was controlled by a motorized delay stage. The pump pulses were chopped by a synchronized chopper at 500 Hz and the change in absorbance was calculated with two adjacent probe pulses (pump-blocked and pump-unblocked). All experiments were performed at room temperature.

Density functional theory (DFT) method was performed to optimize the geometries of the S_0_ and the T_1_ structures of the proposed luminescence units. Time-dependent functional (TDDFT) method was used to estimate the excited state energy levels. The DFT and TDDFT calculations were carried out with Gaussian 09 package at the level of M06-2X/6-31G(d,p). The SOC coefficients were quantitatively estimated at the level of M06-2X/6-31G(d,p) by pySOC program.

## Supplementary information

Supplementary Information for Confined-domain Crosslink-enhanced Emission Effect in Carbonized Polymer Dots

Supporting Movie for CPDsCH3-1

Supporting Movie for CPDsCH3-2

Supporting Movie for CPDsCH3-3
