# Supplementary material for: Confined-domain crosslink-enhanced emission effect in carbonized polymer dots
Source: Light Sci Appl. 2022 Mar 10;11:56. doi: 10.1038/s41377-022-00745-4 (PMC8913797; doi:10.1038/s41377-022-00745-4)
Supplement: Supplementary file 1 — Supplementary Information for Confined-domain Crosslink-enhanced Emission Effect in Carbonized Polymer Dots [file 41377_2022_745_MOESM1_ESM.doc]

**Supplementary Information for**

Confined-domain Crosslink-enhanced Emission Effect in Carbonized Polymer Dots

Songyuan Tao^1^, Changjiang Zhou^2^, Chunyuan Kang^1^, Shoujun Zhu^1,3^, Tanglue Feng^1^, Shi-Tong Zhang^1^, Zeyang Ding^1^, Chengyu Zheng^1^, Chunlei Xia^1^, and Bai Yang^1^,*

^1^ State Key Laboratory of Supramolecular Structure and Materials, College of Chemistry, Jilin University, Changchun, 130012, China.

^2^ College of Chemical Engineering, Zhejiang University of Technology, Hangzhou, 310014, China.

^3^ Key Laboratory of Organ Regeneration and Transplantation of the Ministry of Education, the First Hospital of Jilin University, Changchun, 130061, China.

E-mail: [byangchem@jlu.edu.cn](mailto:byangchem@jlu.edu.cn)

**Table of Content**

1. **Figure S1.** Optical images of CPDs.
2. **Figure S2.** TEM images of CPDs.
3. **Figure S3.** AFM images of CPDs.
4. **Figure S4.** PL decay spectra of CPDs in solution state.
5. **Table S1.** PL lifetime fittings of CPDs in solution state.
6. **Figure S5.** PL spectra of CPDs in solid state.
7. **Figure S6.** PL decay spectra of CPDs in solid state.
8. **Table S2.** PL lifetime fittings of CPDs in solid state.
9. **Figure S7.** Phosphorescence decay spectra of CPDs at 77 K.
10. **Figure S8.** High-resolution XPS spectra of CPDs.
11. **Table S3.** XPS element content of CPDs.
12. **Table S4.** Element analysis of CPDs.
13. **Figure S9.** ^1^H NMR spectra of CPDs.
14. **Figure S10.** ^13^C NMR spectra of CPDs.
15. **Figure S11.** HR-TEM images of CPDsCH_3_-3.
16. **Figure S12.** TGA curves of CPDs.
17. **Figure S13.** TA spectra of CPDs at indicated delay time.
18. **Figure S14.** Kinetic traces of CPDs at different probe wavelengths.
19. **Table S5.** TA kinetic fittings of CPDsCH_3_-1.
20. **Table S6.** TA kinetic fittings of CPDsCH_3_-2.
21. **Table S7.** TA kinetic fittings of CPDsCH_3_-3.
22. **Table S8.** Calculated energy levels of the simplified luminescent units in CPDs.
23. **Figure S15.** RTP decay spectra of CPDsCH3 with different contents of methyl groups.
24. **Table S9.** RTP lifetime fittings of CPDs with different contents of methyl groups.
25. **Figure S16.** RTP lifetimes and QYs of CPDs with different contents of methyl groups.
26. **Table S10.** Calculated k_r_ and ∑k_nr_ values of CPDs.

**
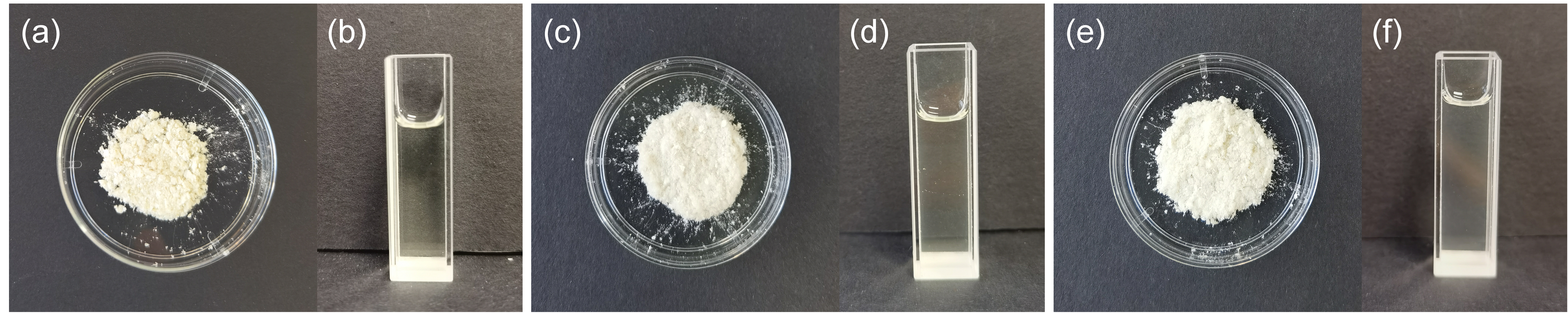
**

**Figure S1.** Optical images of (a) powder and (b) solution of CPDsCH_3_-1, (c) powder and (d) solution of CPDsCH_3_-2, (e) powder and (f) solution of CPDsCH_3_-3.

**Figure S2.** TEM images (inset: size distribution) of (a) CPDsCH3-1, (b) CPDsCH3-2, and (c) CPDsCH3-3.

**
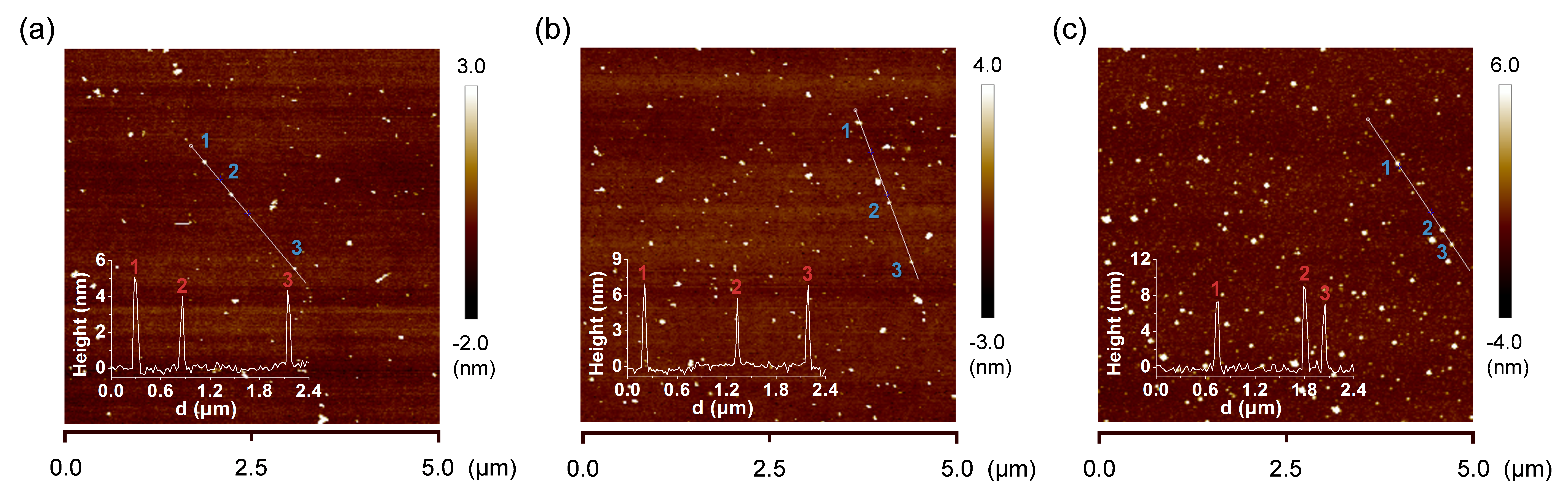
**

**Figure S3.** AFM images (inset: height profiles along the marked line) of (a) CPDsCH_3_-1, (b) CPDsCH_3_-2, and (c) CPDsCH_3_-3.

**
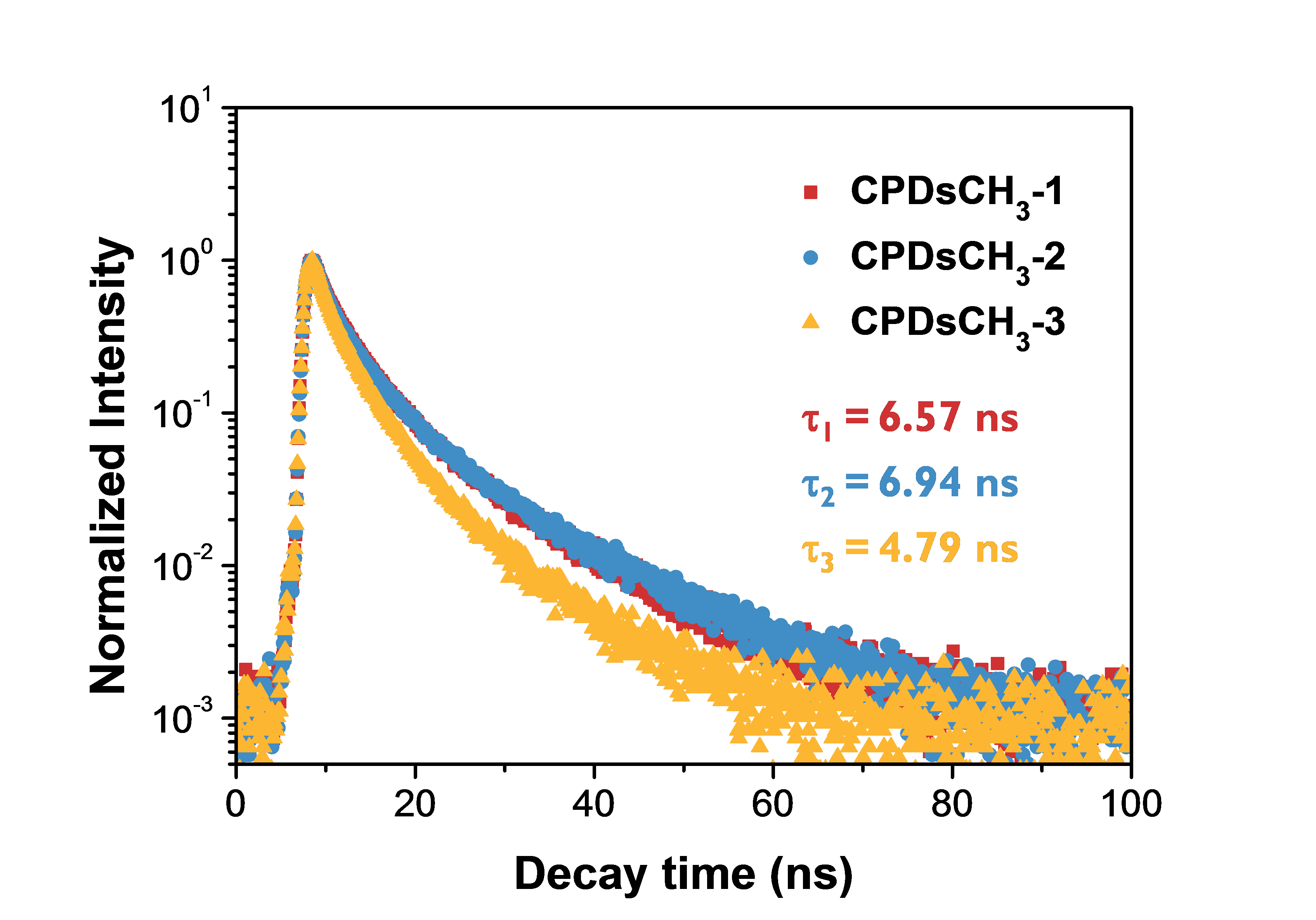
**

**Figure S4.** PL decay spectra of CPDs in solution state.

**Table S1.** PL lifetime fittings of CPDs in solution state.

**
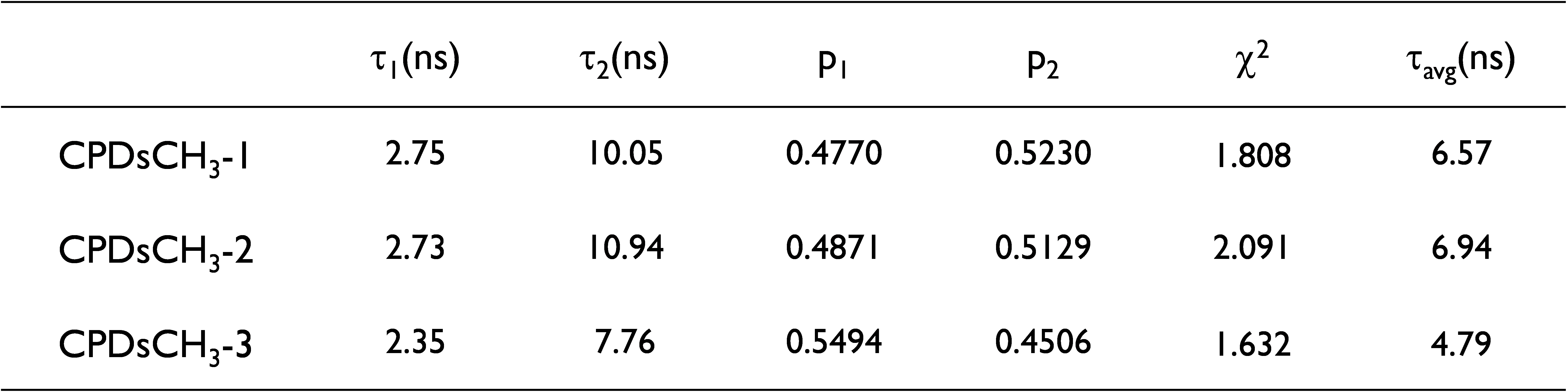
**


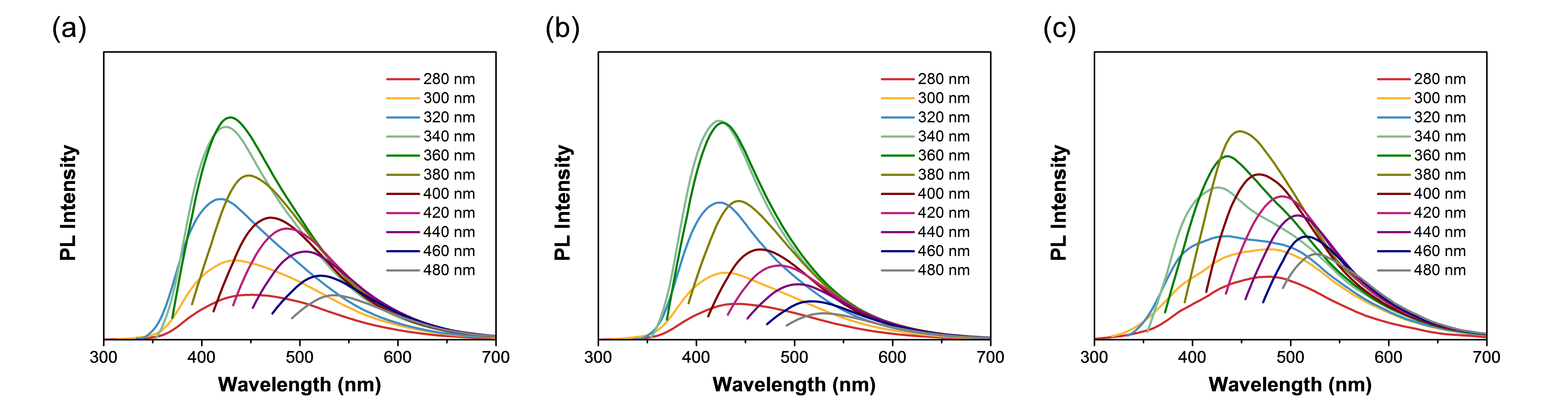


**Figure S5.** PL spectra of (a) CPDsCH3-1, (b) CPDsCH3-2, and (c) CPDsCH3-3 in solid state.

**
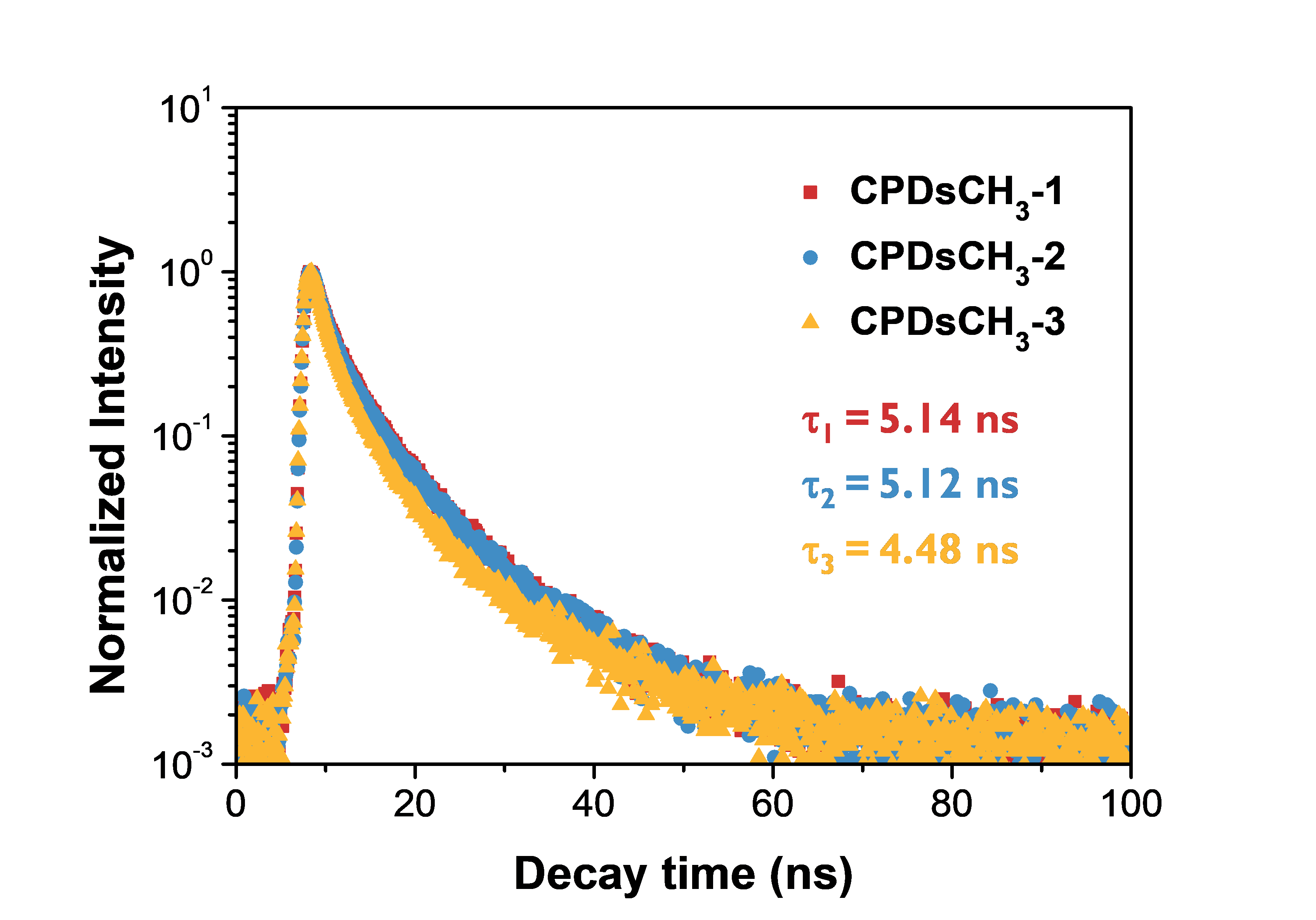
**

**Figure S6.** PL decay spectra of CPDs in solid state.


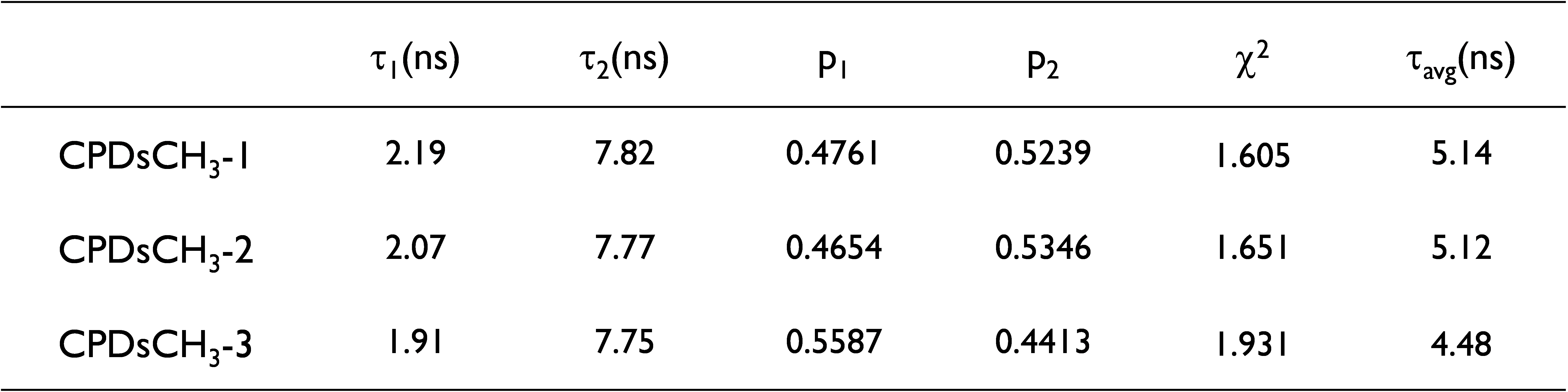
**Table S2.** PL lifetime fittings of CPDs in solid state.

**
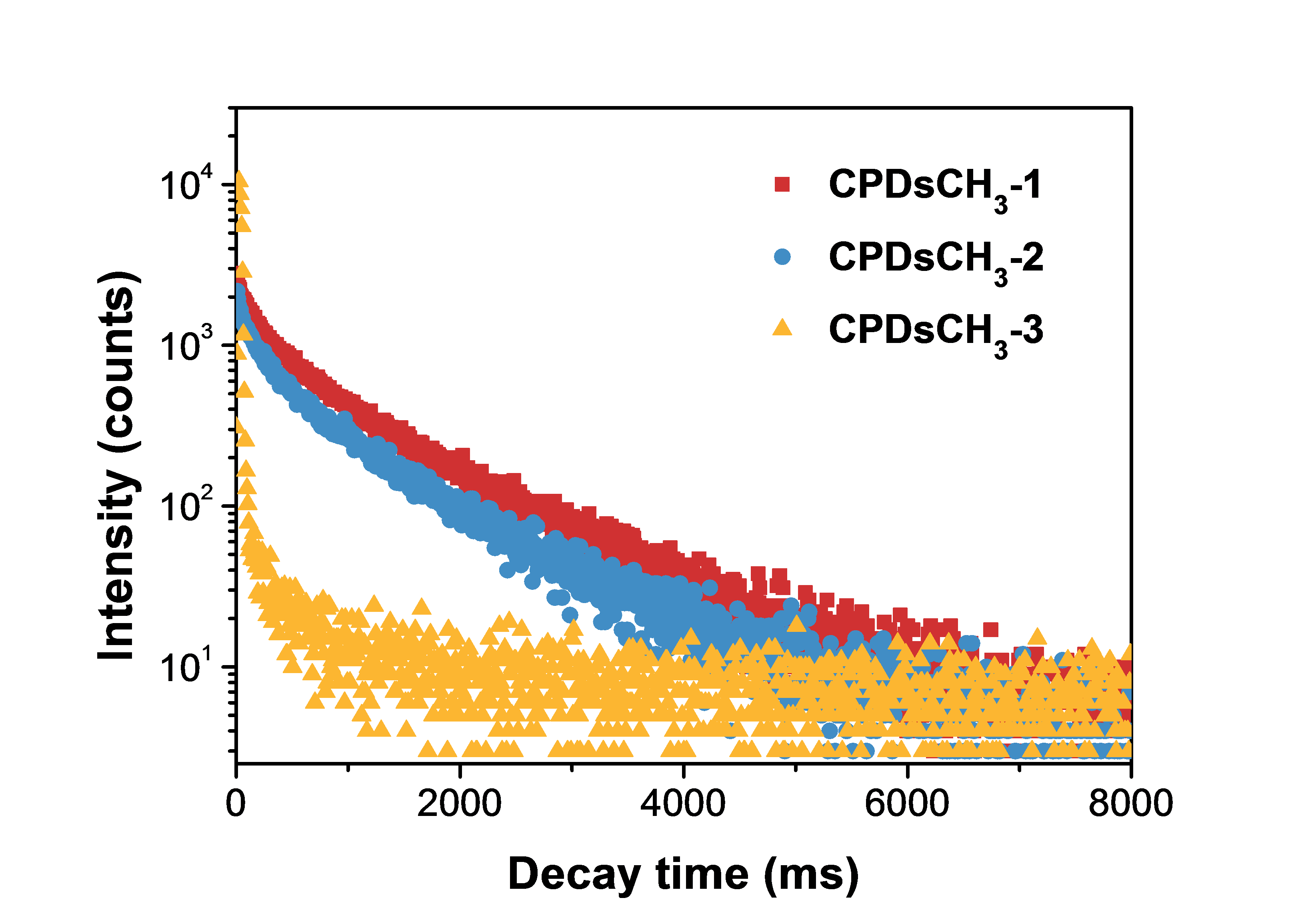
**

**Figure S7.** Phosphorescence decay spectra of CPDs at 77 K.

**
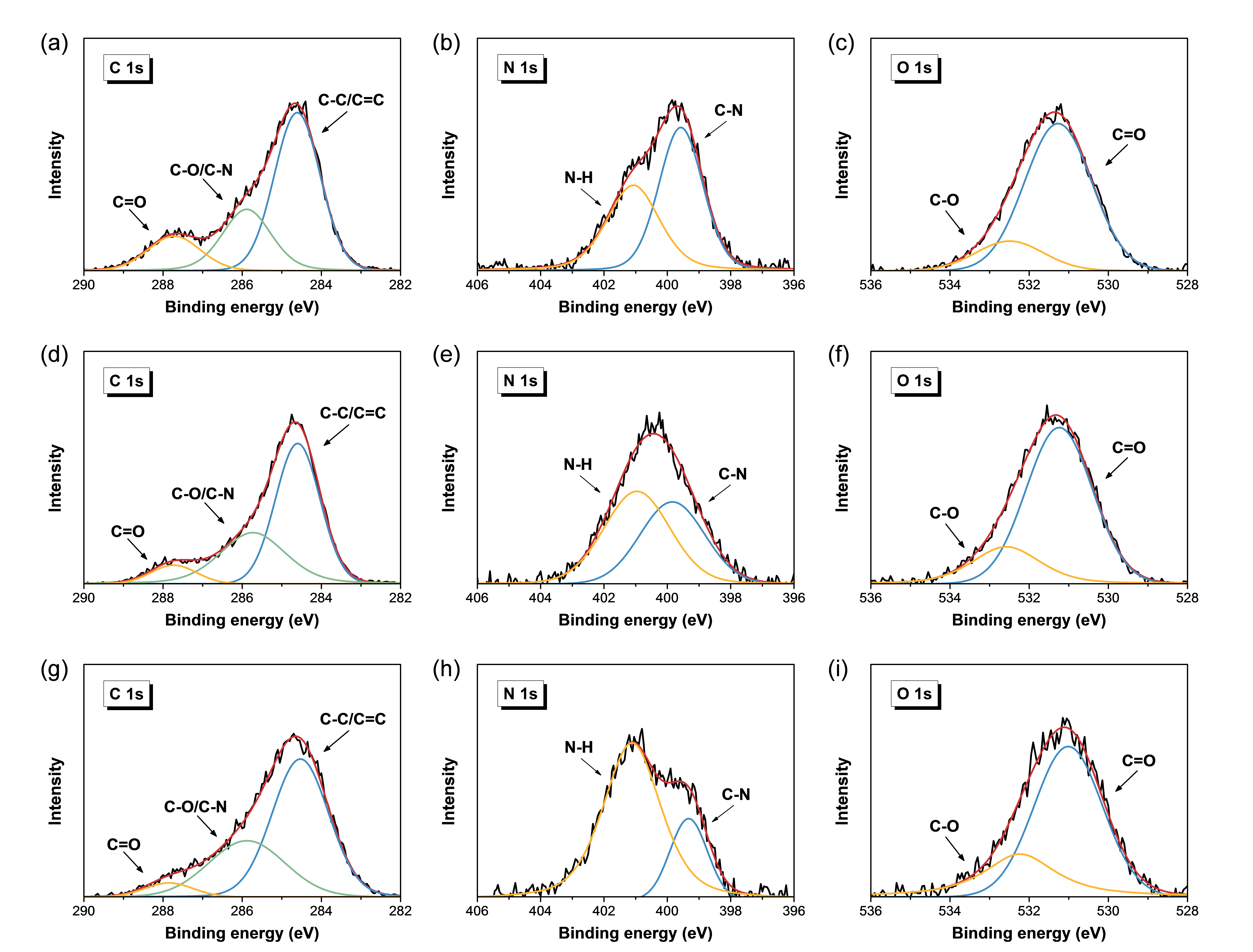
**

**Figure S8.** High-resolution XPS spectra of (a) C 1s, (b) N 1s, and (c) O 1s of CPDsCH_3_-1, (d) C 1s, (e) N 1s, and (f) O 1s of CPDsCH_3_-2, and (g) C 1s, (h) N 1s, and (i) O 1s of CPDsCH_3_-3

**Table S3.** XPS element content of CPDs.

**
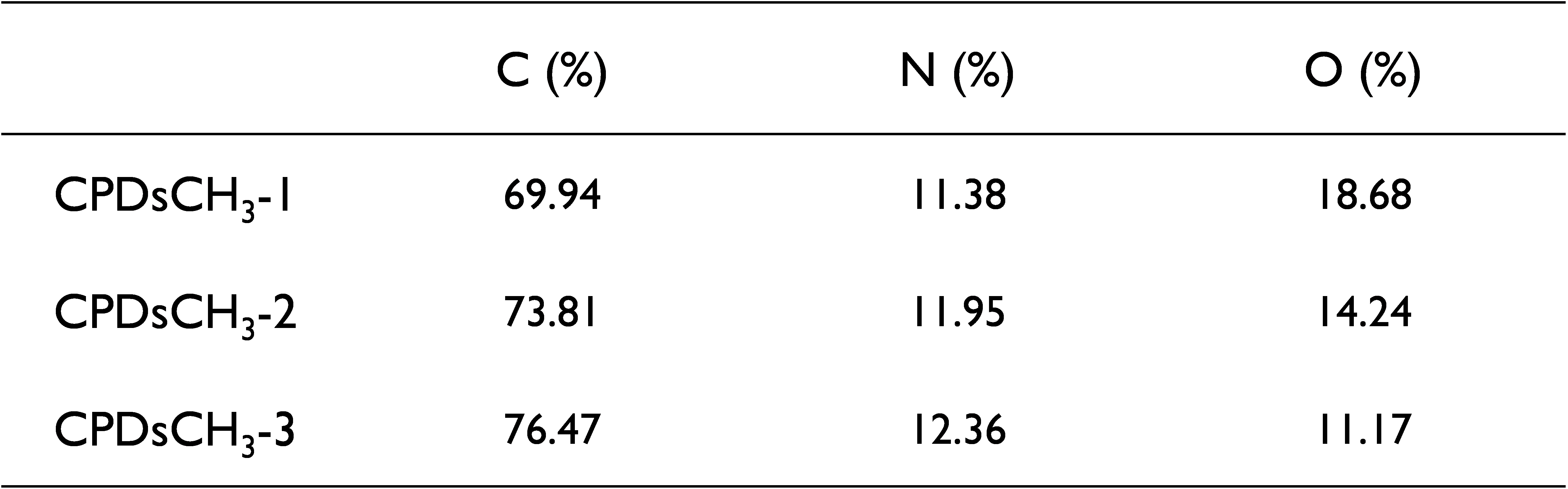
**

**Table S4.** Element analysis of CPDs.

**
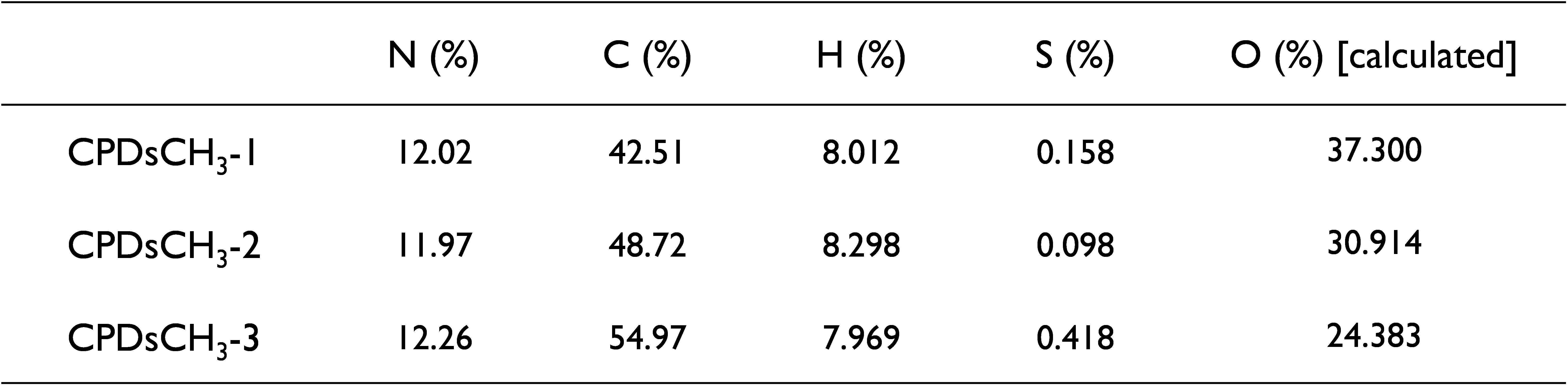
**

**
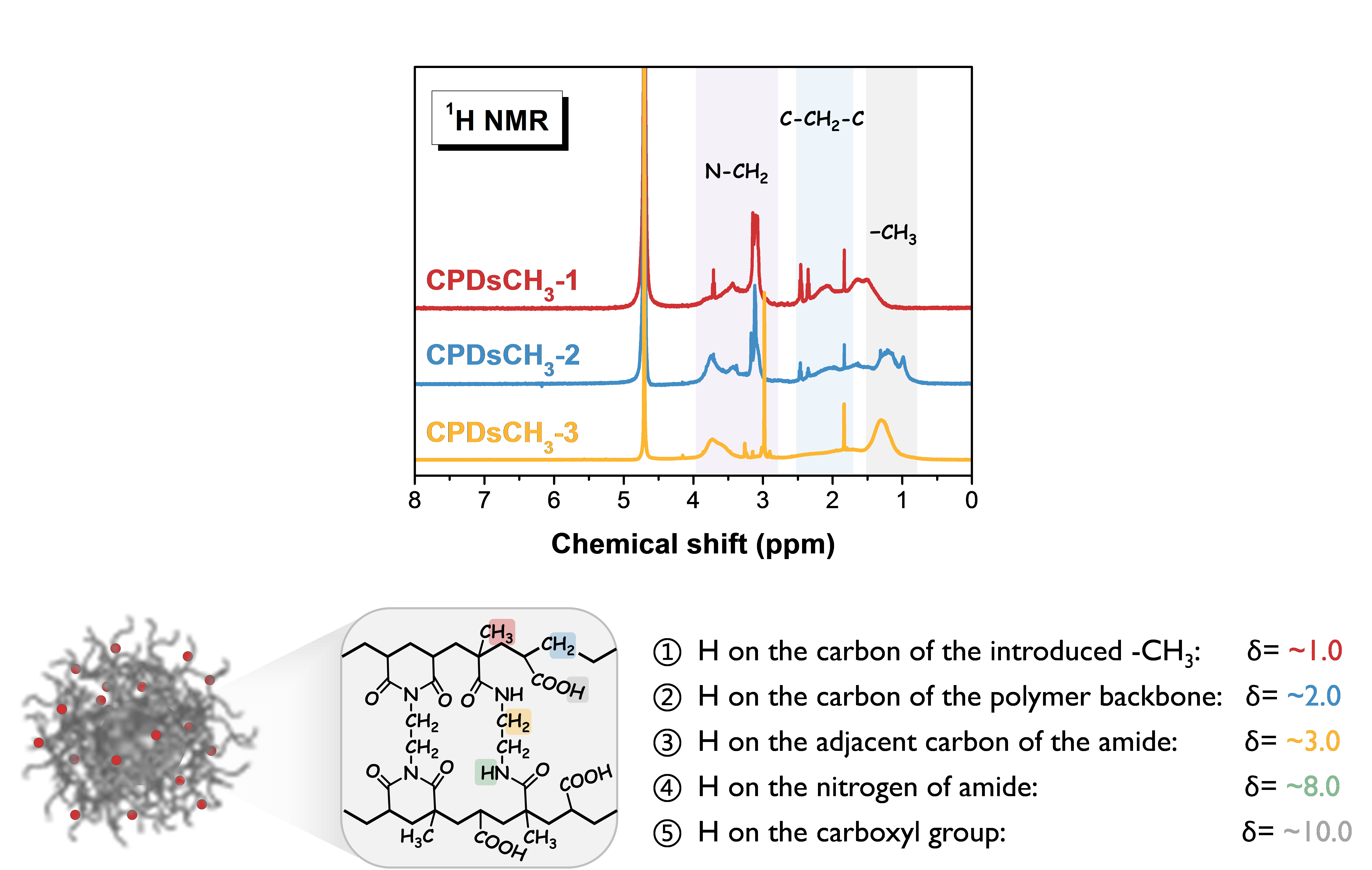
**

**Figure S9.** ^1^H NMR spectra of CPDs (below: theoretical chemical shift of the simplest structure).

**
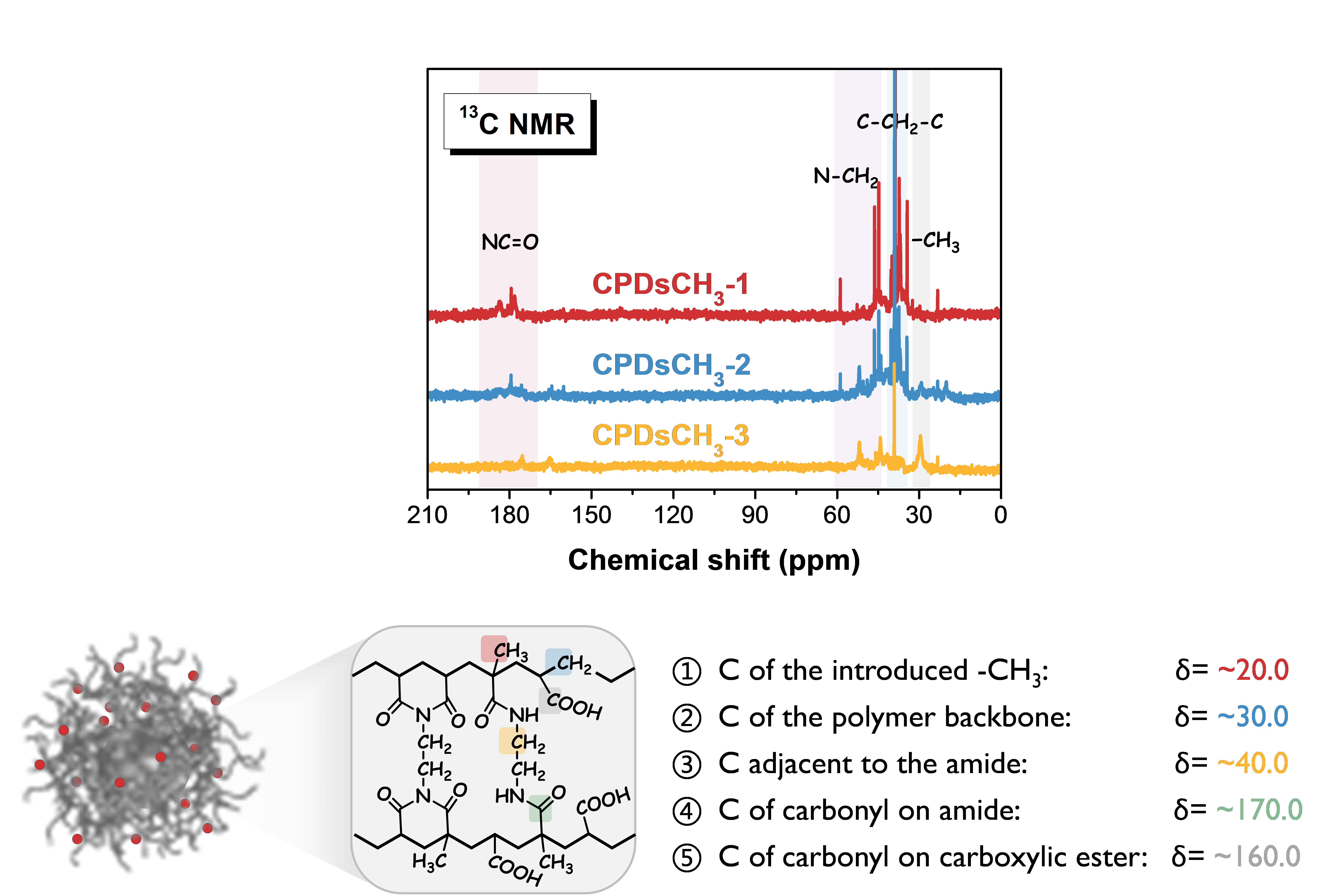
**

**Figure S10.** ^13^C NMR spectra of CPDs (below: theoretical chemical shift of the simplest structure).

**
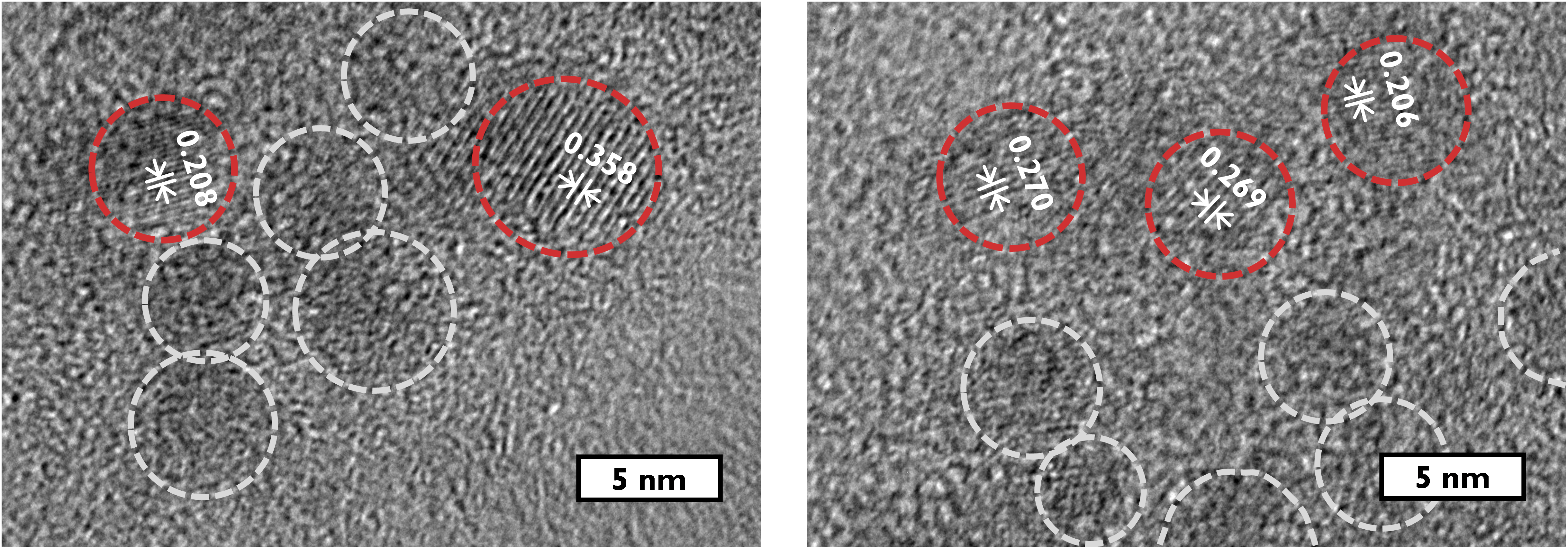
**

**Figure S11.** HR-TEM images of CPDsCH_3_-3 (red circles: CPDs with obvious lattice fringes; gray circles: amorphous CPDs).


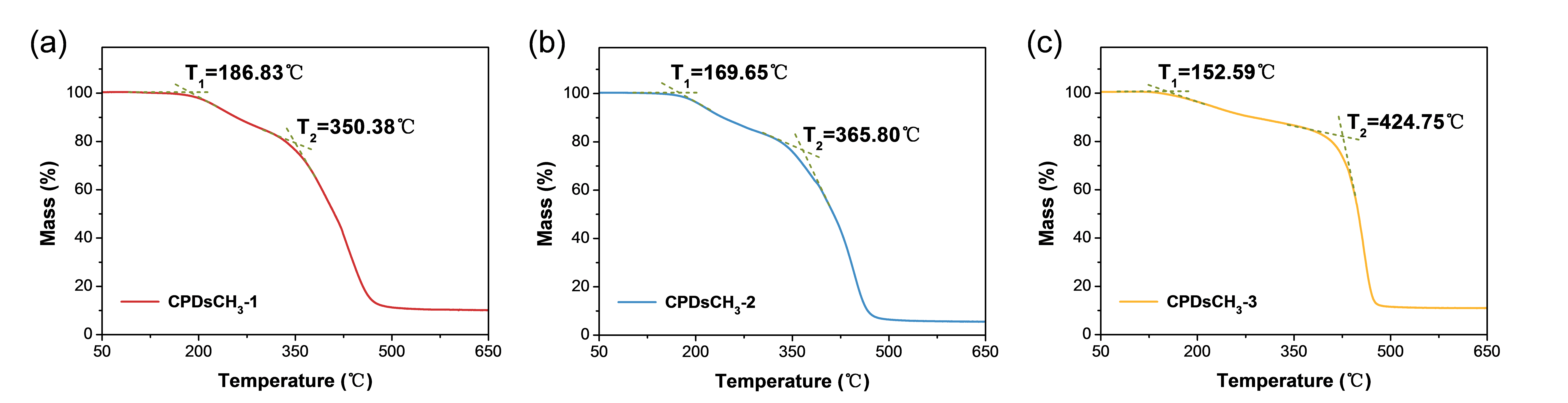


Gray circles: amorphous CPDs).

**Figure S12.** TGA curves of (a) CPDsCH_3_-1, (b) CPDsCH_3_-2, and (c) CPDsCH_3_-3.

**
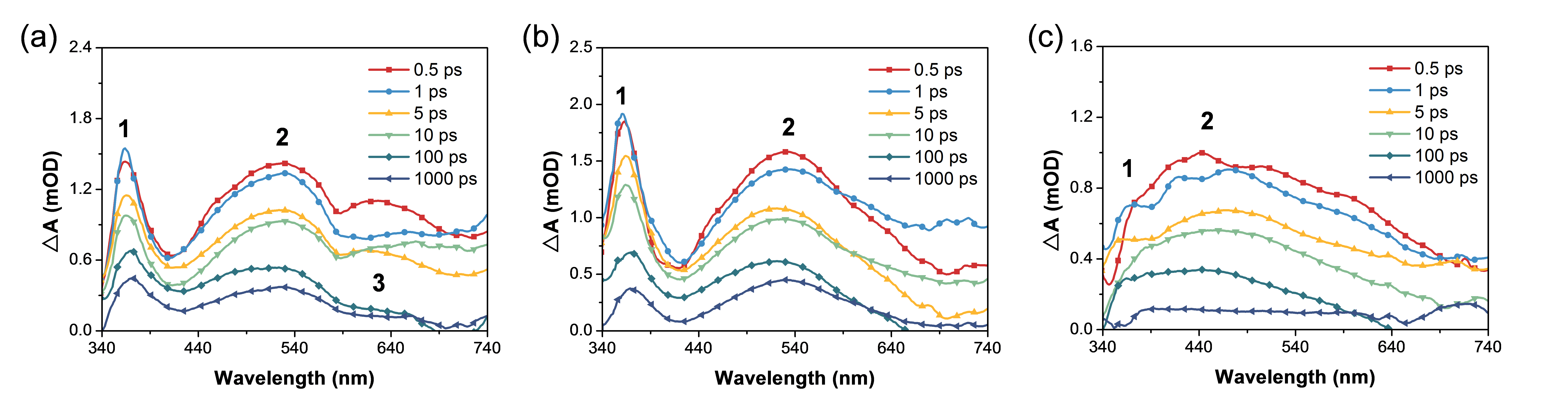
**

**Figure S13.** TA spectra of (a) CPDsCH3-1, (b) CPDsCH3-2, and (c) CPDsCH3-3 at indicated delay time from 0.5 ps to 1 ns.


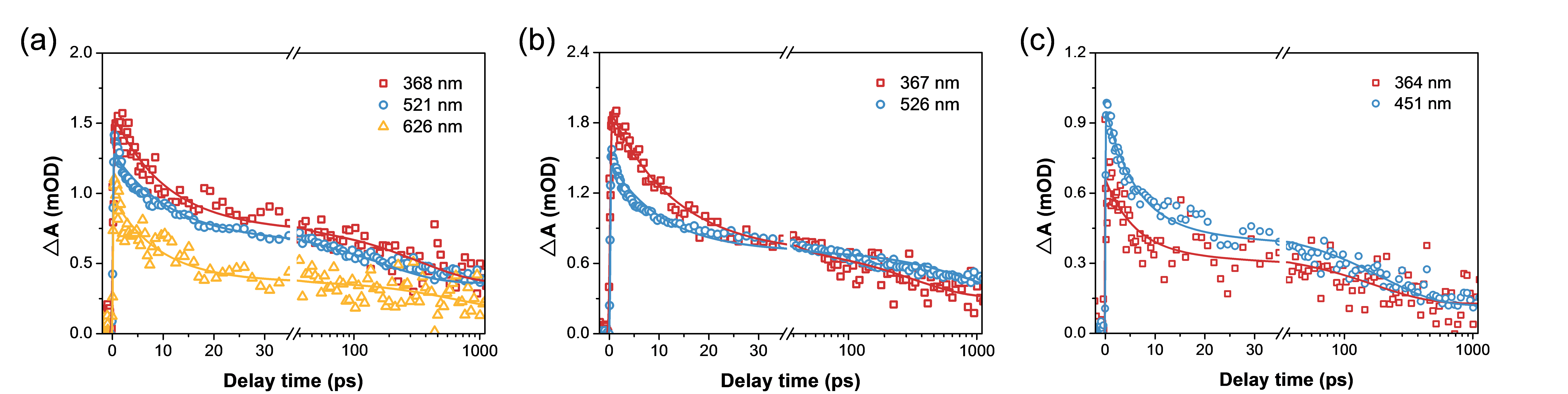


**Figure S14.** Kinetic traces of (a) CPDsCH3-1, (b) CPDsCH3-2, and (c) CPDsCH3-3 at different probe wavelengths (solid lines are fitted curves).

**Table S5.** TA kinetic fittings of CPDsCH_3_-1.

**
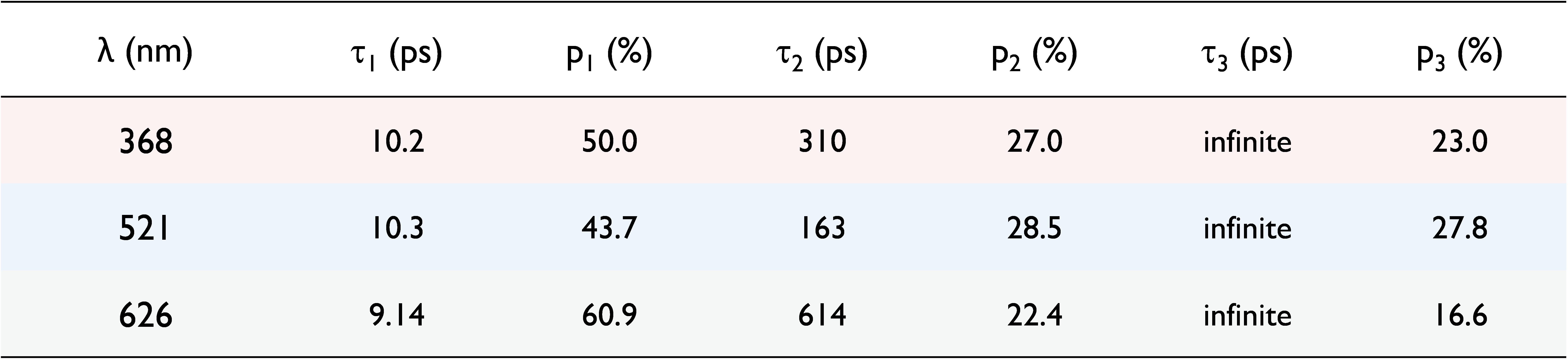
**

**Table S6.** TA kinetic fittings of CPDsCH_3_-2.

**
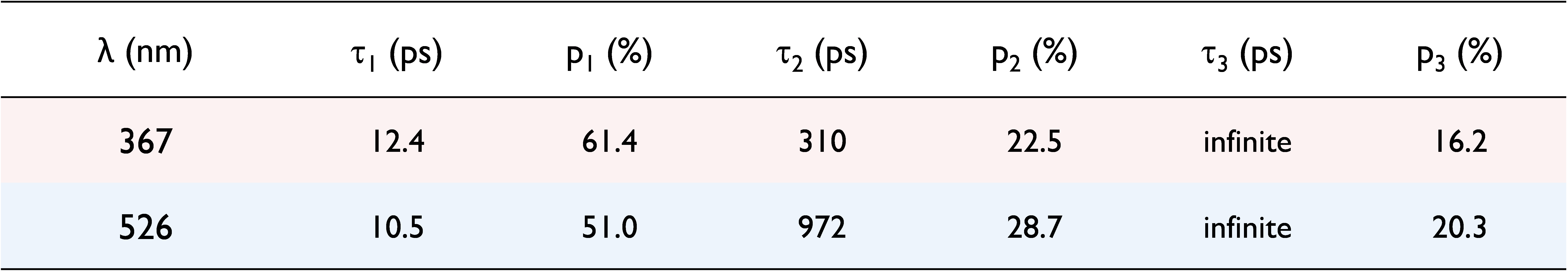
**

**Table S7.** TA kinetic fittings of CPDsCH_3_-3.


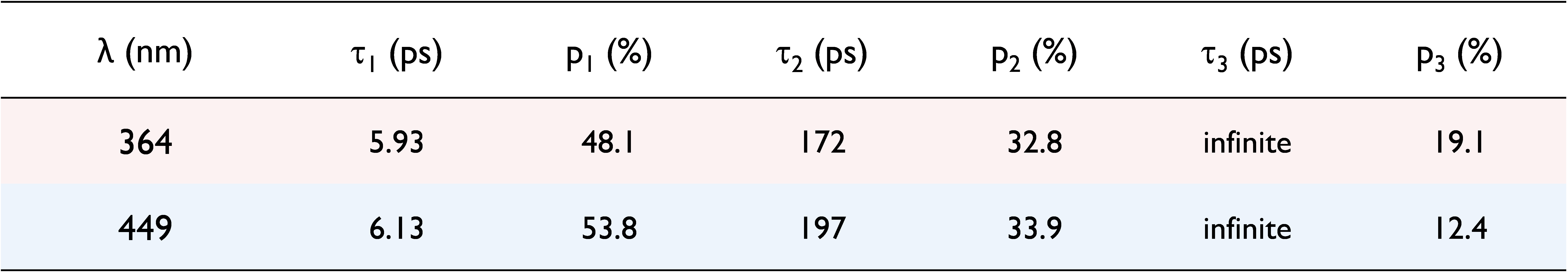


**Table S8.** Calculated energy levels of the simplified luminescent units in CPDs.


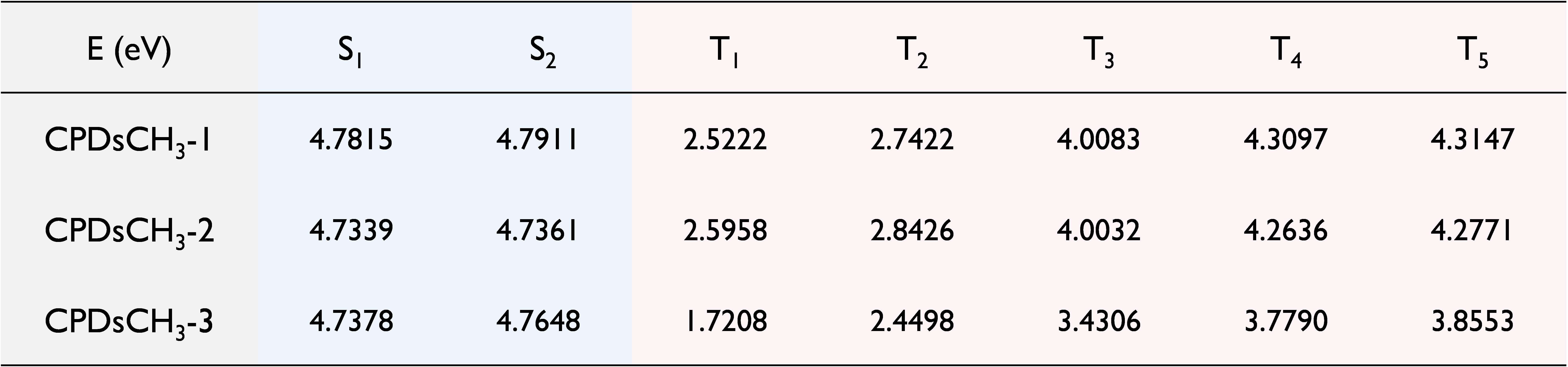


**
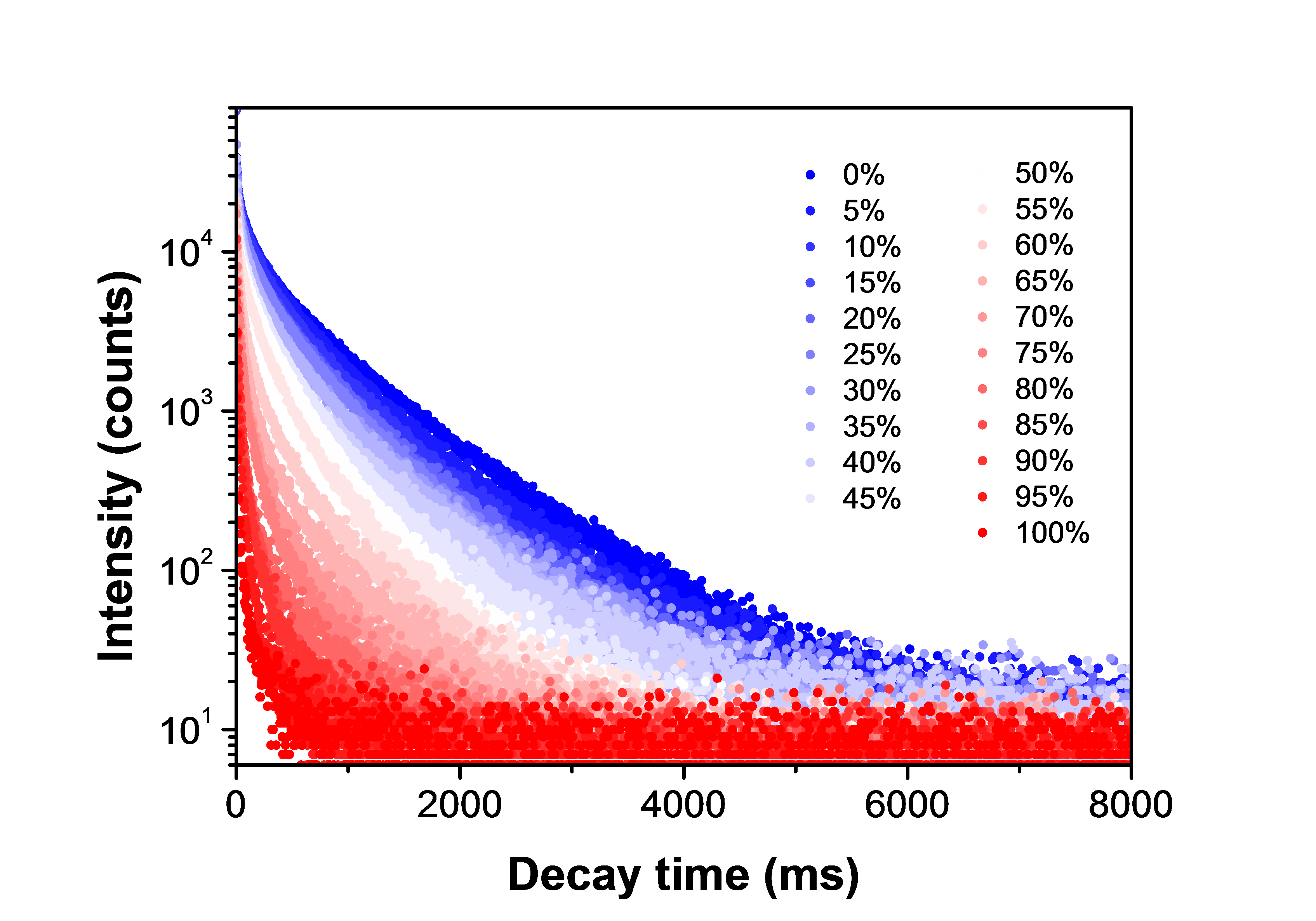
**

**Figure S15.** RTP decay spectra of CPDsCH3 with different contents of methyl groups.

**Table S9.** RTP lifetime fittings of CPDs with different contents of methyl groups.

**
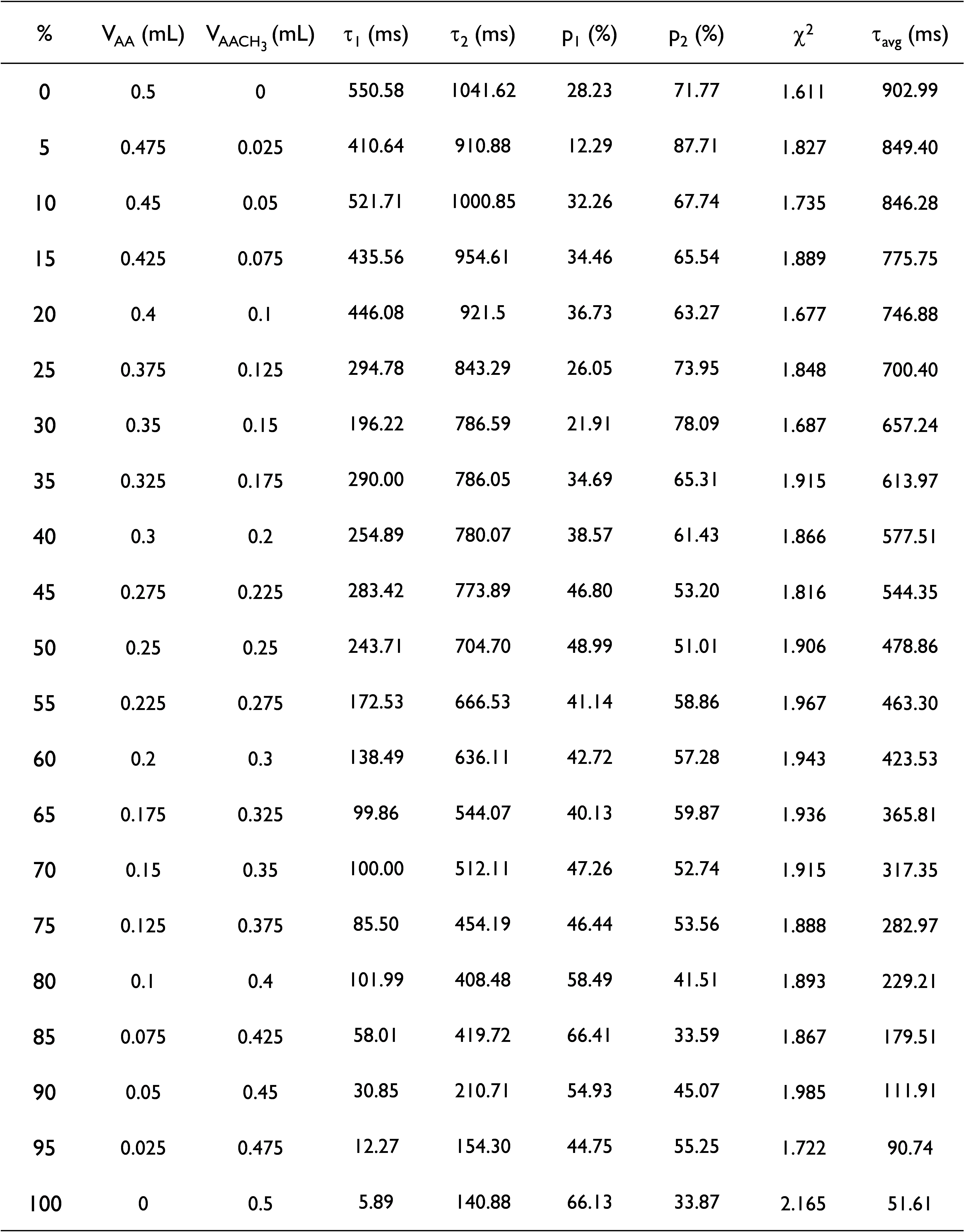
**


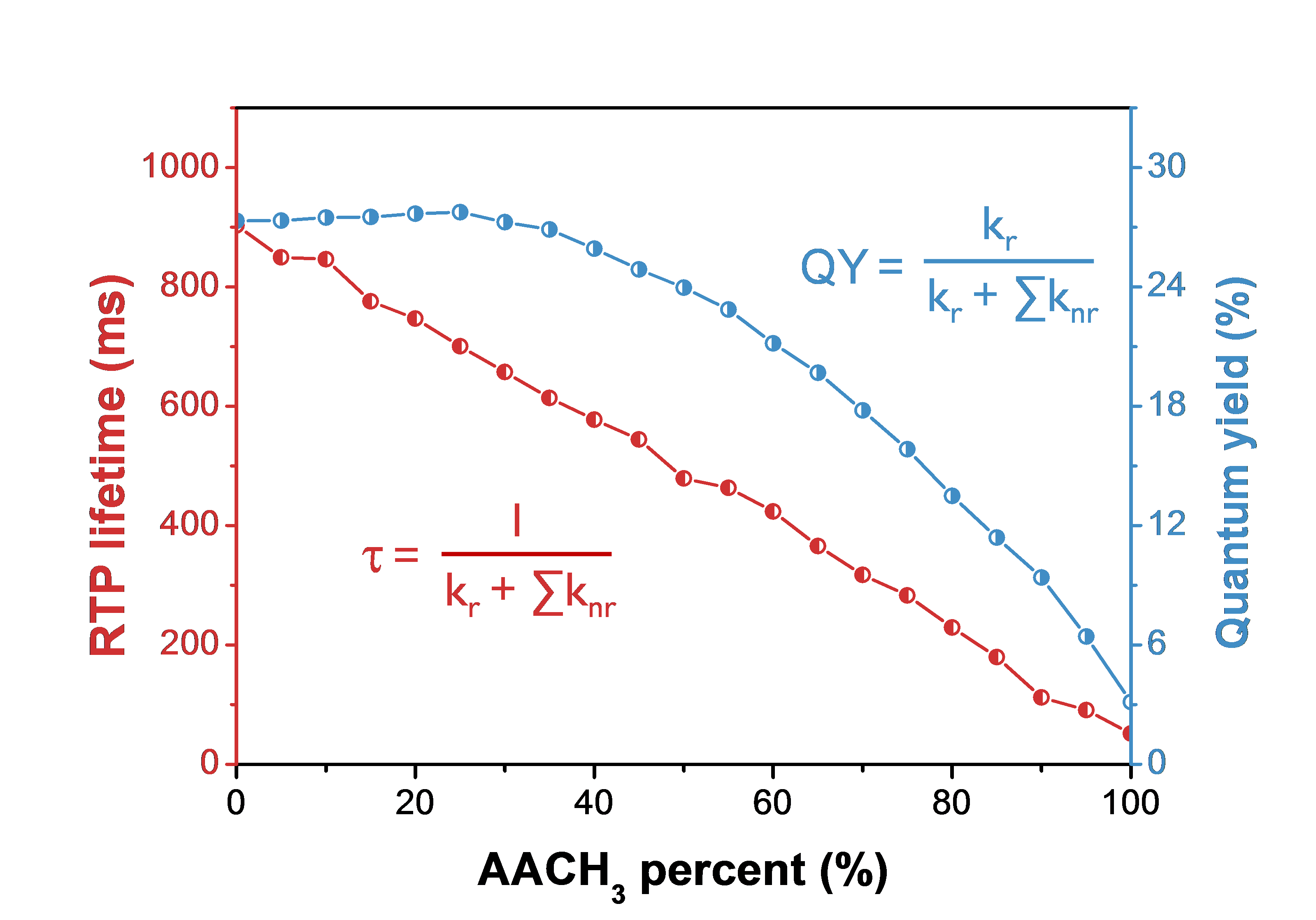


**Figure S16.** RTP lifetimes in solid state and QYs in solution state of CPDs with different contents of methyl groups.

**Note:** Quinine sulfate (0.1 M H_2_SO_4_ as solvent; QY=0.54) was chosen as standard. The excitation wavelength was 360 nm, according to the applicable condition of quinine sulfate. The QYs of CPDs (in water) were calculated by the following equation:

$$\varphi_{x}=\varphi_{st}\cdot\frac{I_{\text{x}}}{I_{st}}\cdot\frac{A_{st}}{A_{x}}$$

Where “ϕ" is the QY, “I” is the measured integrated intensity of PL emission, and “A” is the absorbance value (below 0.1 at 360 nm). The subscript "st" refers to standard with known QY and "x" for the sample.

**Table S10.** Calculated k_r_ and ∑k_nr_ values of CPDs.


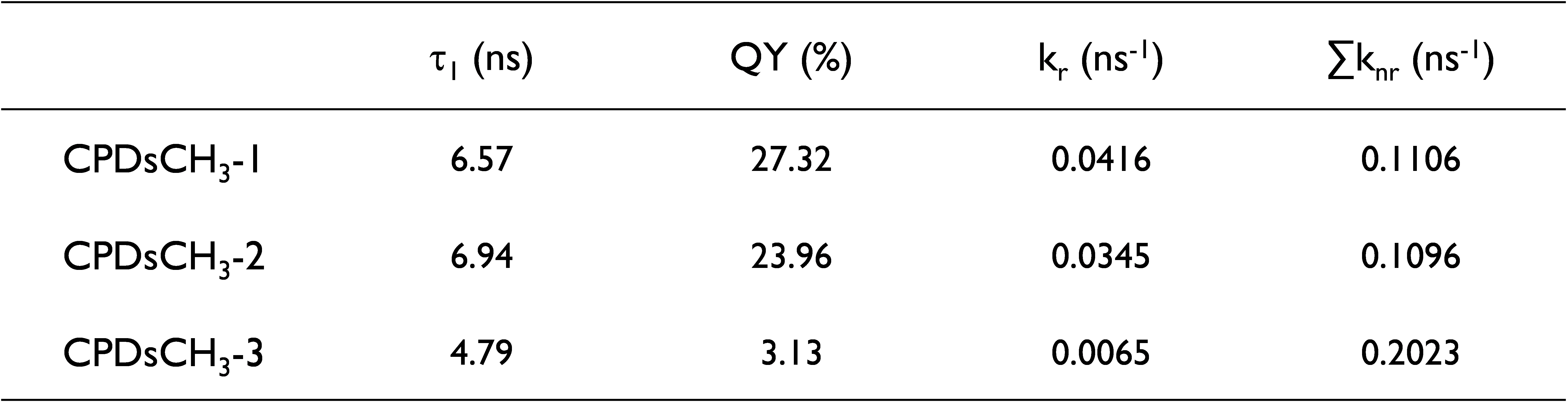


**Note:** a) The values in table were summarized based on the data of CPDs in solution state. b) The corresponding conclusions mainly reflected the transition behaviors of S_1_ state. c) ∑k_nr_ was a comprehensive result of the increased EC and the decreased ISC (∑k_nr_=k_EC_+k_ISC_).
